# Predicting Hepatic Steatosis in Living Liver Donors via Noninvasive Methods

**DOI:** 10.1097/MD.0000000000002718

**Published:** 2016-02-18

**Authors:** Jong Man Kim, Sang Yun Ha, Jae-Won Joh, Dong Hyun Sinn, Woo Kyung Jeong, Gyu-Seong Choi, Geum Youn Gwak, Choon Hyuck David Kwon, Young Kon Kim, Yong Han Paik, Joon Hyeok Lee, Won Jae Lee, Suk-Koo Lee, Cheol Keun Park

**Affiliations:** From the Department of Surgery (JMK, J-WJ, G-SC, CHDK, S-KL); Department of Pathology (SYH, CKP); Department of Medicine, Division of Gastroenterology (DHS, GYG, JHL); and Department of Radiology (WKJ, WJL), Samsung Medical Center, Sungkyunkwan University School of Medicine, Seoul, South Korea.

## Abstract

Hepatic steatosis assessment is of paramount importance for living liver donor selection because significant hepatic steatosis can affect the postoperative outcome of recipients and the safety of the donor. The validity of various noninvasive imaging methods to assess hepatic steatosis remains controversial. The purpose of our study is to investigate the association between noninvasive imaging methods and pathology to detect steatosis in living liver donors and to propose a prediction model for hepatic steatosis.

Liver stiffness measurements (LSMs) and controlled attenuation parameter values in vibration controlled transient elastography, ultrasonography, computed tomography (CT), and magnetic resonance imaging were used as pretransplant screening methods to evaluate living liver donors between 2012 and 2014. Only 1 pathologist assessed tissue sample for hepatic steatosis.

The median age of the 79 living donors (53 men and 26 women) was 32 years (16–68 years). The CT liver–spleen attenuation (L–S) difference and the controlled attenuation parameter values were well correlated with the level of hepatic steatosis on liver pathology. Multivariate analysis showed that liver stiffness measurement (LSM) (β = 0.903; 95% CI, 0.105–1.702; *P* = 0.027) and the CT L to S attenuation difference (β = −3.322; 95% CI, −0.502 to −0.142; *P* = 0.001) were closely associated with hepatic steatosis. We generated the following equation to predict total hepatic steatosis: Hepatic steatosis = 0.903 × LSM – 0.322 × CT L to S attenuation difference (AUC = 86.6% and *P* = 0.001). The values predicted by the equation correlated well with the presence of hepatic steatosis (*r* = 0.509 and *P* < 0.001).

The combination of nonenhanced CT L to S attenuation difference and transient elastography using vibration controlled transient elastography provides sufficient information to predict hepatic steatosis in living liver donor candidates.

## INTRODUCTION

Most hepatic steatosis or liver disease in Western countries is commonly diagnosed to nonalcoholic fatty liver disease (NAFLD). Recent studies reported that the incidence of NAFLD in Korea has also increased because of Westernized lifestyles and increasing body mass index (BMI).^1–3^

The assessment of hepatic steatosis is essential for living liver donor selection because significant hepatic steatosis can affect postoperative outcomes in the donor. In addition, the development of primary dysfunction, early allograft dysfunction, poor overall graft survival, and other complications have been reported in recipients of steatotic grafts in liver transplantation.^4,5^

Surgeons usually perform hepatic steatosis assessment during living donor surgery, but this method is very subjective and difficult. The utilization of steatotic living liver donors has been highly variable among surgeons in different medical centers and in different countries. Therefore, an accurate diagnosis of hepatic steatosis and objective quantification of this condition is of paramount importance for the clinical decision making regarding liver donation by live donors, the safety of the living donor, and for estimation of recipient prognosis.

Although various biochemical, anthropometric, and radiological methods have been extensively evaluated, the current gold standard for the diagnosis and assessment of hepatic steatosis severity is liver biopsy.^6–8^ However, liver biopsy can only be performed in selected subjects and is not suitable for screening or for monitoring changes in hepatic steatosis during the evaluation of living liver donors. This is due to the biopsy's invasiveness, expense potential for sampling error, potential intra- and interobserver variability during pathological interpretation, and the potential for serious complications.^7–9^

To overcome these limitations, several noninvasive imaging techniques have been proposed to assess hepatic steatosis in living liver donors, including ultrasonography (US), computed tomography (CT), and magnetic resonance imaging (MRI).^10–15^ Additionally, controlled attenuation parameters (CAP) and liver stiffness measurements (LSMs) based on the properties of US signals acquired by vibration controlled transient elastography (VCTE) have recently been introduced, based on the postulate that fat affects ultrasound propagation.^16^ Unfortunately, these tools are limited by cost, restricted availability, operator dependence, and poor sensitivity.^7,17^ In addition, the ability of noninvasive imaging methods to reliably determine hepatic steatosis is controversial.

Thus, the purpose of our study is to investigate the association between noninvasive methods and pathological steatosis in living liver donors and to propose a model to predict hepatic steatosis using noninvasive methods.

## PATIENTS AND METHODS

### Study Population

This was a retrospective study approved by our institutional review board (SMC 2015–07–147). The institutional review board waived the need for informed consent for the study, but written informed consent was obtained from all subjects for every procedure. Between 2012 and 2014, 79 consecutive living donors who underwent VCTE (Fibro-Scan; Echosens, Paris, France), US, CT, and MR evaluation for liver donation within four weeks of living donor hepatectomy were identified using the radiologic information system and pathology database at a single transplant center.

### Evaluation and Selection of Living Liver Donors

Live liver donation was based on a strict volunteer process. Living donor evaluations, such as, radiologic evaluations and laboratory examinations, were described previously.^18^ Psychiatric assessment was routinely performed. During the initial screening of the potential living liver donors, subjects with alcohol use ≥40 g/wk, the presence of serum hepatitis B surface antigen, hepatitis C virus antibodies, or antibodies to human immunodeficiency virus were excluded.

All donors were examined with abdominal Doppler US to evaluate liver quality, including evaluation for hepatic steatosis. Abdominal CT imaging was performed to assess vascular anatomy and calculate liver volume to ensure adequate liver graft and remnant liver volume after hepatectomy.^18^ An estimated graft volume greater than 40% of the recipient's standard liver volume was considered acceptable. Donors were excluded if there was an indication of severe fatty change on US or unfavorable hepatic parenchymal, vascular, or biliary morphology prior to the operation. Our living donor liver transplantation (LDLT) program limited the extent of donor hepatectomy to 70% of the entire liver volume. Finally, MRI was performed to verify biliary anatomy when eligibility was confirmed.

Absolute exclusion criteria included any inoperable hepatic vascular variations and any underlying medical condition that increased perioperative risk. The upper age limit was 55 years for right and extended right lobe graft donors. Older patients more than 55 years of age were sometimes highly selected based on their medical conditions. Liver donation was permitted if the donor livers had no more than 30% steatosis.^18,19^ When pretransplant imaging showed evidence of severe fatty liver, preoperative liver biopsy was performed. No autologous blood was preserved.

### Ultrasonography Evaluation

For US evaluation, patients were placed in the supine or left lateral decubitus position, and sonographers acquired serial abdominal images using the subcostal and intercostal approaches.^20,21^ US was performed on fasting living liver donors. The sonographers, who had more than 7 years of clinical experience, were blinded to patient clinical features. The degree of hepatic steatosis based on liver echogenicity was evaluated and classified as none, minimal, mild, moderate, or severe. The overall assessment of liver echogenicity was determined based on the sonographic difference between the hepatic parenchyma and the adjacent kidney.^20–23^

### Computed Tomography Evaluation

For all donor candidates, we performed nonenhanced and enhanced CT with a multidetector row helical scanner to detect abnormal lesions, possible malignancies, and to assess vascular anatomy. One radiologist, who was blinded to the pathological and clinical results, retrospectively reviewed the CT imaging. For each scan, 3 regions of interest were obtained from each segment. We obtained liver and spleen densities in Hounsfield units (HU) and used the CT liver to spleen (L–S) attenuation difference to assess steatosis.^24^ To obtain the indices, hepatic attenuation was measured by averaging the HU of 2 1.5 × 1.5 cm square regions of interest in 8 segments.^25^ Median splenic attenuation was also calculated by 3 random attenuation measurements on 3 transverse sections at different splenic levels. Large vessels and artifacts were avoided.

### Magnetic Resonance Imaging Evaluation

One radiologist, who was blinded to the pathological results, retrospectively reviewed the MRI images and recorded the signal intensity measurements of the liver and spleen. Three regions were checked in the right lobe above and below portal vein, and left lobe, respectively. Upper, mid, and lower portion of spleen were checked for comparing liver.^21–23^ Vessels and artifacts were avoided.

### Liver Stiffness and Controlled Attenuation Parameter Measurements

For all patients, assessment of CAP and LSMs were performed within 2 weeks of the surgery, at the pre-transplant evaluation. Only one experienced technician, who was blinded to the patient’ clinical data, performed the LSMs. LSMs using VCTE were performed on the right lobe of the liver through the intercostal space with the patient in the dorsal decubitus position with the right arm in maximal abduction.^26^ The tip of the transducer probe was placed on the skin between the ribs over the right lobe of the liver. At this site, the distance between the skin and liver capsule (skin-capsular distance) was measured, and an attempt was made to collect ≥10 valid LSMs.

The liver stiffness results are expressed as kilopascals. The median value of the successful measurements was selected to represent the patient's liver stiffness value. The CAP measures ultrasonic attenuation in the liver at 3.5 MHz using signals acquired by the M probe based on VCTE.^3^ The CAP is calculated only when LSM is valid for the same signals, ensuring that one obtains liver ultrasonic attenuation simultaneously and in the same volume of liver parenchyma as the LSM. The final CAP value was the median of the individual CAP values, using the same valid measurements, and was expressed in dB/m.

### Donor Biopsy and Histologic Assessment

All living liver donors underwent wedge resection in liver segment 4 during the transplant surgery in order to assess hepatic steatosis. Resected liver tissues were immediately frozen, cut into 5 μm thick slices, and stained with hematoxylin–eosin to assess for hepatic steatosis. Frozen sections were analyzed by pathologists blinded to the results of noninvasive assessment.^11^ If greater than 30% macrosteatosis or microsteatosis were identified in the frozen section, the living donor operation was discontinued and the abdominal wall was closed. All living donor liver samples were made placed in a paraffin block to detect hepatic steatosis.^27,28^ All biopsies were examined by 1 liver pathologist blinded to the imaging findings. The severity of macrosteatosis or microsteatosis was estimated under 20× magnification and reported.

### Statistical Analysis

Continuous variables were expressed as medians with ranges, and categorical variables were expressed as numbers and percentages. Wilcoxon rank sum tests were used to assess differences between continuous variables. Correlations were analyzed between LSMs, CAP, US grading, MRI grading, and CT L–S attenuation differences. Multivariate analysis was performed using linear regression analysis. Receiver operating characteristic curves were generated to identify and predict hepatic steatosis. All statistical analyses were performed with SPSS 21.0 software. All reported *P* values were 2-sided, and *P* values <0.05 were considered statistically significant.

## RESULTS

### Donor Patients

Of the 79 donors, 53 (67.1%) were men and 26 (32.9%) were women. The median age was 32 years (range: 18–68 years). No patients had hypertension or diabetes. The median BMI was 23.1 (range: 17.7–34.2). None of the patients underwent liver biopsy for screening in the pretransplant period. Additionally, none of the 79 donors had more than 30% macrosteatosis or microsteatosis on frozen section or histology. The median degree of both macrosteatosis and microsteatosis was 1% (range: 0%–15%). The median total amount of hepatic steatosis was 2% (range: 0%–25%). The number of patients with greater than 10% macrosteatosis, microsteatosis, or total hepatic steatosis were 3 (3.8%), 4 (5.1%), and 8 (10.1%), respectively. Accordingly, all living liver donors underwent hepatectomy.

For the donor operations, 69 patients underwent right hemihepatectomy, 3 underwent left hemihepatectomy, and 7 underwent left lateral sectionectomy. The median operative time was 387 minutes (range: 243–632 minutes). The median graft-to-recipient weight ratio was 1.00 (range: 0.60–3.55), and the median hospitalization time was 13 days (range: 8–36 days).

### Evaluation Correlation

**FIGURE 1:**
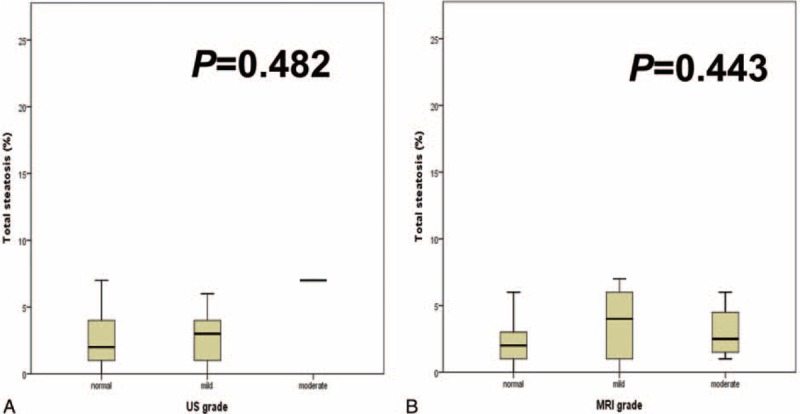
Hepatic steatosis according to (A) ultrasonography and (B) magnetic resonance imaging grade.

**FIGURE 2:**
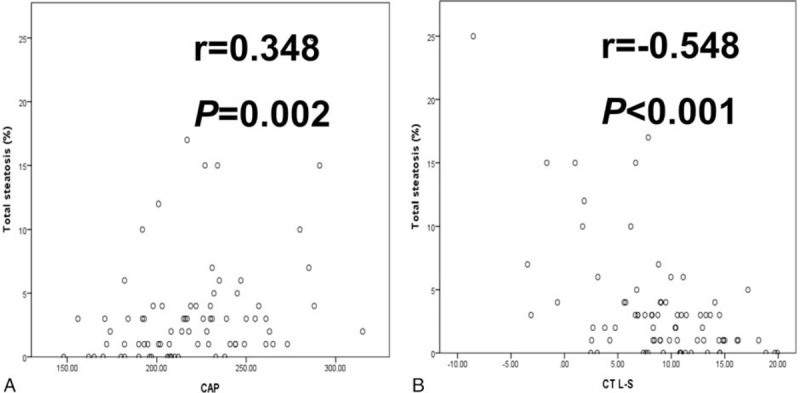
Correlation between hepatic steatosis and controlled attenuation parameter and unenhanced computed tomography liver–spleen attenuation difference values.

Macrosteatosis was well correlated with microsteatosis (*r* = 0.572 and *P* < 0.001). LSM, US, and MRI findings were not associated with the level of hepatic steatosis (Figure 1). However, the CT L–S attenuation difference and CAP were well correlated with the amount of hepatic steatosis in the biopsy samples (Figure 2).

### Predicting Hepatic Steatosis

**FIGURE 3:**
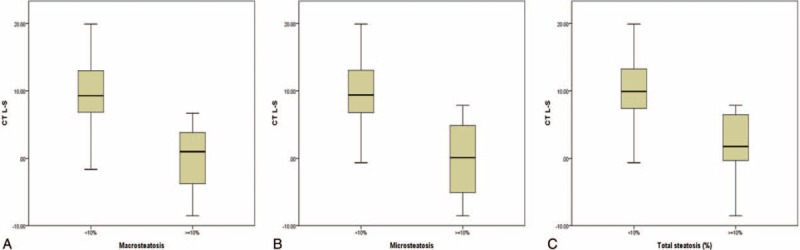
Hounsfield units according to 10% hepatic steatosis.

**FIGURE 4:**
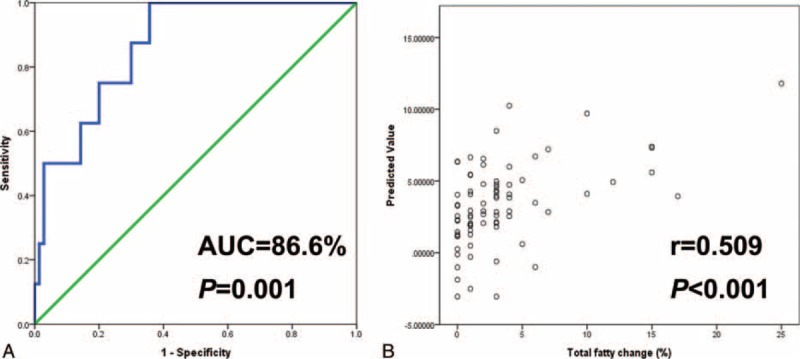
A, Receiver operating characteristics curve. B, Predictive values of the equation.

Multivariate analysis showed that CT L–S attenuation differences were closely associated with macrosteatosis and microsteatosis in pretransplant living liver donors (Table 1). We analyzed the HU of the CT L–S attenuation differences according to the hepatic steatosis cut-off of 10% (Table 2 and Figure 3). These figures indicate that a CT L–S attenuation difference greater than 10 HU may be associated with hepatic steatosis of less than 10%. However, multivariate and receiver operating characteristic curve analyses revealed that a CT L–S attenuation difference greater than 10 HU was not associated with macrosteatosis, microsteatosis, or total hepatic steatosis of less than 10%. LSMs (β = 0.903; 95% CI, 0.105 to 1.702; *P* = 0.027) and CT L–S attenuation differences (β = −3.322; 95% CI, −0.502 to −0.142; *P* = 0.001) were closely associated with hepatic steatosis. We generated the following equation to predict total hepatic steatosis: Hepatic steatosis = 0.903 × LSM – 0.322 × CT L to S attenuation difference (AUC = 86.6% and *P* = 0.001). The predicted values from the equation were well correlated with hepatic steatosis (*r* = 0.509 and *P* < 0.001) (Figure 4).

**TABLE 1:**
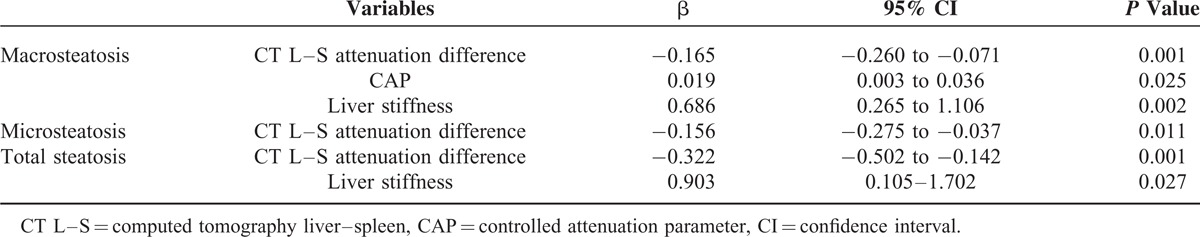
Multivariate Analysis Revealed that Unenhanced Computed Tomography Liver–Spleen Attenuation Difference Was Closely Associated With Hepatic Steatosis

**TABLE 2:**
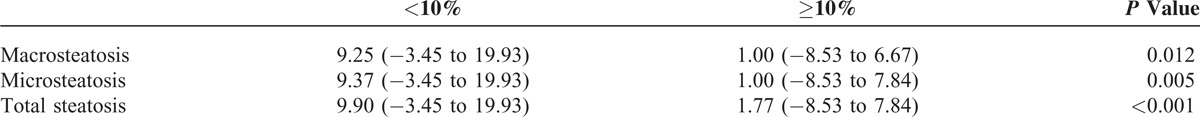
Nonenhanced Computed Tomography Hounsfield Units According to the Cutoff Value of 10% Hepatic Steatosis

## DISCUSSION

Healthy volunteers are able to donate a partial liver graft to compatible patients with end-stage liver disease or hepatocellular carcinoma, known as LDLT. In Korea, partial major liver resection, including right hemihepatectomy, has induced severe morbidity and mortality in living liver donors. Thus, the selection of suitable living liver donors is very important to ensure safe, successful graft procurement, and successful LDLT for the recipients, as well. The most serious ethical concern for LDLT is the risk to the healthy donor, who will undergo a major operation without any potential health benefit. Therefore, it is important to preserve donor health and to exclude unsuitable candidates from donation. Thus, an accurate quantitative assessment of hepatic steatosis in donors is crucial for the proper selection of liver grafts.

Fatty liver is the most common diffused liver disease that precludes an outwardly healthy patient from being a donor. Specifically, the increasing prevalence of NAFLD in the general population poses a risk for organ donation. For example, the first case of living liver transplantation donor death, reported in Japan, was of a donor with nonalcoholic steatohepatitis.^29^ Although 30% steatosis has been considered an appropriate upper threshold for living donors, it is not a fixed or generalizable value, and some institutions favor more conservative cutoffs.^5^ The current dilemma in living donor selection is whether to include grafts with >10% steatosis. Therefore, it is important to determine the relative diagnostic accuracies of different imaging modalities according to various threshold levels of hepatic steatosis.

Liver biopsy is the current gold standard for assessing hepatic steatosis in living liver donors, but biopsy is rarely performed because of its invasiveness. Not to mention, liver biopsy includes a reported 20% incidence of pain,^7^ and the procedures associated with living liver donation may be unpleasant to donors. Considering that donors are healthy individuals, many centers, including ours, perform biopsies selectively. Furthermore, lack of homogeneity in fat distribution raises the question of whether a single biopsy specimen can accurately assess hepatic steatosis. Thus, numerous imaging modalities have been evaluated for predicting the degree of hepatic steatosis, but none of the modalities have been ideal candidates.^7^ Therefore, it is important to predict which individuals will benefit most from a liver biopsy.

Overall, our results showed that the studied CAP, CT, and MRI indices performed similarly in estimating liver fat content in our selected population of liver donors. However, multivariate analysis suggested that only CT L–S attenuation differences and LSMs were closely associated with hepatic steatosis in living donors. Our study results suggest that the combined use of CT L–S attenuation differences and LSMs provide the ability to detect hepatic steatosis on imaging.

A recent study reported that severe hepatic steatosis influenced LSMs in patients with NAFLD, which revealed that false-positive LSM result rates were strongly influenced by the presence of severe hepatic steatosis.^30^ In addition, another study reported that the combined presence of diabetes and steatosis was associated with high liver stiffness.^31^ The present study shows the usefulness of transient elastography in detecting mild hepatic steatosis.

Ultrasonography is the simplest imaging method for detecting and characterizing hepatic steatosis, and thus, has commonly been used to screen for fatty liver disease. It offers a fairly accurate diagnosis of moderate-to-severe hepatic steatosis (≥30%), with a reported sensitivity ranging from 81.8% to 100.0% and a specificity as high as 98%.^23,32^ However, US has low sensitivity for mild-to-moderate steatosis, and thus, might fail to detect early-stage disease. Overall, the sensitivity of US in detecting hepatic steatosis is unsatisfactory, and it is even less effective at diagnosing mild hepatic steatosis.^33^ Our study also confirmed these limitations of US in evaluating mild hepatic steatosis.

Magnetic resonance imaging is considered to be the most accurate noninvasive method for quantifying fat content of the liver.^15,17^ A recent study showed that a combination of MR fat quantification and MR elastography can provide 100% sensitivity and a 100% negative predictive value for the detection of substantial steatosis or fibrosis (≥F1) in liver donor candidates.^15^ However, the complexity of these procedures makes them difficult to use in routine organ procurement. The limitations of MRI include variations due to differences between MRI systems, scanning parameters, and methods of analysis. In addition, MRI is relatively expensive and can have low diagnostic performance for estimating hepatic fat content in cases with iron deposition.^23^ Our study did not evaluate MR elastography; thus, we cannot validate the combination of MR fat quantification and MR elastography. However, liver stiffness was closely associated with hepatic steatosis in the present study, and thus liver stiffness examination using elastography is essential for the detection of hepatic steatosis.

Our study indicated that nonenhanced CT is the best CT method for estimating hepatic steatosis because of its close association with hepatic steatosis and its ease of measuring liver attenuation. Several studies also reported that this method is highly sensitive (88%–95%) and specific (90%–99%) for the detection of hepatic steatosis.^34^ In addition, nonenhanced CT scans are usually preferred in order to avoid the potential errors of contrast-enhanced CT caused by variations in liver attenuation related to contrast injection methods and scan timing.^23^

The present study showed that the combination of CT and transient elastography could be used as a screening tool to determine the appropriateness of a potential donor liver by means of exclusion. By this approach, potential living donors who were found to have moderate to severe hepatic steatosis on CT imaging and transient elastography could avoid unnecessary biopsy and its associated risks and costs.^7^ Therefore, we propose that CT be used to clinically evaluate potential liver transplantation donors for exclusion based on hepatosteatosis >10% determined via volume analysis and hepatic vascular anatomy assessment.

Our study has some limitations. First, our study population of 79 living liver donors was relatively small. Second, the study population included a large portion of donors with normal livers and low BMI. Moreover, a significant number of patients were excluded predominantly based on US evaluation, and the studied living donors underwent operations. Potential liver donors with high level of steatosis were excluded, and only 8 patients (10.1%) with greater than 10% hepatic steatosis were included. Therefore, this study had biased data. Third, our study assumed a homogeneous distribution of hepatic steatosis. However, a wedge liver biopsy with a small sampling volume cannot accurately represent the entirety of the liver, because the distribution of hepatic steatosis is heterogeneous, which might limit the relevance of correlations between histologic fat quantification and imaging. Fourth, since it was a retrospective single-center study, the generalizability of our findings to other patient populations requires confirmation.

In conclusion, a combination of nonenhanced CT liver-spleen attenuation difference and transient elastography can provide sufficient information for the detection of hepatic steatosis in living liver donor candidates, potentially reducing the necessity of liver biopsy and increasing donor and recipient safety.
